# Bilingual Language Development in Infancy: What Can We Do to Support Bilingual Families?

**DOI:** 10.1177/23727322211069312

**Published:** 2022-02-23

**Authors:** Laia Fibla, Jessica E. Kosie, Ruth Kircher, Casey Lew-Williams, Krista Byers-Heinlein

**Affiliations:** 1 5618 Concordia University, Montreal, Quebec, Canada; 2 6740 Princeton University, Princeton, NJ, USA; 3 Mercator European Research Centre on Multilingualism and Language Learning Fryske Akademy, Leeuwarden, the Netherlands

**Keywords:** infancy, children, bilingualism, dual language learners, language acquisition, language experience, language input, language outcomes

## Abstract

Many infants and children around the world grow up exposed to two or more languages. Their success in learning each of their languages is a direct consequence of the quantity and quality of their everyday language experience, including at home, in daycare and preschools, and in the broader community context. Here, we discuss how research on early language learning can inform policies that promote successful bilingual development across the varied contexts in which infants and children live and learn. Throughout our discussions, we highlight that each individual child's experience is unique. In fact, it seems that there are as many ways to grow up bilingual as there are bilingual children. To promote successful bilingual development, we need policies that acknowledge this variability and support frequent exposure to high-quality experience in each of a child's languages.

## Tweet

Bilingual infants benefit from high-quality, high-quantity experience in their languages. Policies tailored to each community, like paid parental leave and bilingual community/library programs, can support families in transmitting their languages. [link_to_paper]

## Key Points

- Many infants and young children around the world grow up bilingual.
- There is substantial variability in the experience of bilingual children, including who speaks different languages around them and how the languages are used.
- Growing up bilingual does not cause deficits in language learning. However, bilingual children with language deficits might need support in each of their languages.
- Language learning is linked to the quantity and quality of language infants and children hear, so policies that promote bilingualism should enable infants and children to receive sufficient high-quality experience in each of their languages.
- Policies must be adapted to each child's context to be able to support diverse family structures across the globe.

Many infants around the world grow up bilingual, meaning they receive regular exposure to two or more languages (Wei, 2000). Early language development predicts later language skills, cognitive abilities, and school achievement (e.g., Hurtado et al., 2014; Scarborough et al., 2009; Schwab & Lew-Williams, 2016), and early bilingualism is linked to benefits across many different domains (e.g., Bialystok, 2018). Many bilingual infants—particularly those with high levels of support in each language—grow up to be highly proficient speakers of their languages. However, in some cases, bilingual children have lower proficiency in one of their languages (Fillmore, 1991). Supporting families and communities is essential for ensuring positive language outcomes in bilingual children—defined as families see fit—so that bilingual children can benefit maximally from their unique language environments.

Here, we bring together scientific findings from bilingual language acquisition to highlight the role of caregivers as well as the sociocultural environment in fostering bilingual infants’ journeys toward reaching their full potential. In doing so, we acknowledge that each bilingual experience is unique—there are nearly as many ways to grow up bilingual as there are bilingual children. Contexts for bilingualism may be quite different across families, daycares and preschools, communities, and cultures (Rowe & Weisleder, 2020), and research to date does not yet reflect the full range of bilingual experience. Taking this diversity into account is key to understanding early bilingual development. We conclude by articulating policy implications for better supporting infants and their families, based on established findings from research on early bilingual—as well as monolingual—language learning.

## Who Is Bilingual?

In the past, the term “bilingual” has been used to refer to individuals who speak two languages just as proficiently as monolinguals who speak one language (e.g., Peal & Lambert, 1962). However, given that this represents only a tiny minority of those who use two or more languages, researchers currently employ the term much more inclusively, extending it those all who use two or more languages in everyday life (Byers-Heinlein & Lew-Williams, 2013). In this paper, we define bilingual infants as those who have regular exposure to more than one language from early in development.

Bilingualism is common and increasing in many parts of the world (Wei, 2000). It is prevalent in regions of many continents, including Australia, Africa (e.g., Senegal and South Africa), North America (e.g., Canada), Asia (e.g., India and the Philippines), and Europe (e.g., Switzerland and Belgium). An estimated 15% of children in Australia (Australian Bureau of Statistics, 2006), 18–25% of children in Canada (Schott et al., 2021), 29% of children in parts of Spain (Statistical Institute of Catalonia, 2007), 90% of children in Singapore (Wu et al., 2020), and 23–43% of children in parts of the United States (U.S. Census Bureau, 2019) grow up bilingual (with some variation across regions and children's ages). In many contexts, bilingual populations are systematically different from monolingual populations beyond the number of languages spoken; for example bilingualism is often associated with migrants and/or minoritized groups (Extra & Verhoeven, 1999).

Each bilingual infant's language learning experience is unique: children differ in terms of the specific languages they are learning, when they start learning each, and how they encounter them. Consequently, each bilingual child will have their own developmental path (De Bruin, 2019). For example, some bilingual infants hearing similar languages may be able to transfer syntactic and lexical knowledge from one language to the other (Floccia et al., 2018), while those learning dissimilar languages will have to learn different rules for each. Bilingual children also differ in terms of the age at which they begin learning each language: some hear their languages from birth from their primary caregivers, while others hear certain language(s) only at home and are exposed to an additional language somewhat later in development when they enter childcare. Further, circumstances can change across an infant's life, impacting how much and from whom they hear each of their languages (e.g., Prevoo et al., 2011).

A common factor across bilingual language development is the crucial role of the quantity and quality of exposure infants receive in their different languages (Marchman et al., 2010; Ramírez-Esparza, et al., 2017). For example, we would not expect a child to learn a language they only hear for 5 min per day (low quantity) or to learn a language from 5 h a day of isolated vocabulary words recited from an audiotape (low quality). It is difficult to know exactly what combination of quantity and quality is required, but some studies estimate that children need a minimum of 10–25% of overall exposure in a language to achieve fluency in it, which will only occur if their language experience is rich and varied (e.g., Place & Hoff, 2011). If children do not receive sufficient high-quality bilingual exposure, they are unlikely to become proficient in all their languages.

## Myths About Early Bilingualism

A commonly-held concern is that early exposure to multiple languages is confusing. To the contrary, young infants can perceive the difference between different languages within the first few days or months of life (Bosch & Sebastián-Gallés, 2001; Byers-Heinlein et al., 2010). The early years (approximately birth to age 3) represent a sensitive period for language acquisition, when the brain is particularly receptive to the properties of language (Werker & Tees, 2005), and where children can accumulate large amounts of experience with their languages. For this reason, bilingual children exposed to their languages earlier in development become better at them, and will speak and process them in a more native-like manner (Bialystok, 2001). Exposure to a second language should thus not be unnecessarily delayed, and should begin as soon as feasible. Nonetheless, many individuals who begin learning a new language after the sensitive period become highly proficient speakers, particularly when they are motivated to learn (Dörnyei, 2009).

The notion that bilingualism causes language disorders and delays is also false: these occur in bilingual children at the same rates as in monolingual children (Paradis et al., 2011). In fact, many children with various developmental disabilities also grow up bilingual, including children with specific language impairment (Paradis et al., 2011), children with Down Syndrome (Kay-Raining Bird et al., 2005), and children with autism spectrum disorder (Gonzalez-Barrero & Nadig, 2018). Bilingualism does not appear to be detrimental to children with developmental deficits in language or cognition, and in fact, bilingualism could be a boost in some cases. For example, for children with autism, bilingualism could expand their possibilities for social interactions (Peristeri et al., 2021) and even mitigate difficulties related to shifting between tasks (Gonzalez-Barrero & Nadig, 2019).

## Bilingual Language Experience From Caregivers

Regardless of whether they are developing typically or atypically, children's caregivers play a key role in providing crucial language experience (Casillas et al., 2020; De Houwer, 2007; Tsinivits & Unsworth, 2021). For example, the amount of infant-directed speech (Ramírez-Esparza et al., 2014) and turn-taking that children experience (Donnelly & Kidd, 2021) predicts their speech and language development. Different families approach bilingualism in different ways. In some, each caregiver speaks only one language to their infant (the “one-person-one-language approach”). In others, caregivers speak more than one language to the infant and even a third one between them (Orena et al., 2020). In heritage language families (i.e. families who speak a language other than the one(s) of the wider community), caregivers may use only the heritage language in the home, and employ the community language(s) outside the home (Ballinger et al., 2020). Extended family, whether or not they live with the infant, can also be a rich source of language exposure. The amount of time family members (immediate and extended) as well as others (including friends, neighbors, educators) spend with the child and the languages they speak further shape the unique experience of each bilingual child.

Children benefit from continuous, high-quality language exposure during the first years of life. High quality interactions involve a natural back-and-forth rhythm—even with infants who can only coo and smile in response. Sentences with varied words and structures, which are adapted to the infants’ level, are also characteristic of high-quality language environments. In many (although not all) cultures, high-pitched, slow, melodic, infant-directed speech is typical, and has been shown to support early language learning (Golinkoff et al., 2015). Practices such as shared book reading, where an adult reads to a child, can benefit bilingual language acquisition as well (e.g., Tsybina & Eriks-Brophy, 2010). However, some research suggests that families may tend to emphasize one language over the other in their home literacy practices, leading to unbalanced exposure to each of the child's languages (Gonzalez-Barrero et al., 2021), and parents often lack access to literacy materials in heritage languages and bilingual formats (Ahooja et al., 2021).

One unique type of interaction in bilingual families is code-switching: the use of two or more languages within the same sentence or conversation. The types of code-switching and the reasons to code-switch vary across communities, even across bilingual families. Code-switching is highly associated with parents’ abilities in each language as well as the sociolinguistic environment of the family (Byers-Heinlein & Lew-Williams, 2013). Societal attitudes toward code-switching can sometimes be negative, particularly in monolingual, less diverse environments (Dewaele & Wei, 2014). Parents may wonder whether exposure to code-switching is detrimental to bilingual language development, and indeed, there is some evidence from laboratory-based tasks that infants can sometimes be slightly slower to process code-switches, just as adults (Byers-Heinlein et al., 2017).

At the same time, code-switching is a creative communicative resource (García & Hesson, 2015) that can enhance children's understanding and development. For example, in real-life settings, parents seem to use code-switching in efforts to support successful bilingual language acquisition: their main reasons to code-switch are to bolster their infant's understanding and to teach vocabulary (Kremin et al., 2020). Moreover, as children develop, they begin to produce speech that often includes code-switches, which can be a marker of linguistic competence (Yow et al., 2018). Discouraging bilingual children from code-switching can lead to linguistic insecurity and contribute to negative educational outcomes (García & Tupas, 2018).

## The Role of the Sociocultural Environment

Infants’ exposure to each language is strongly influenced by the sociocultural environment in which they grow up. For example, children growing up in highly bilingual societies receive more consistent exposure across their different daily environments and become more proficient in their languages. By contrast, children growing up in highly monolingual societies often hear one of their languages mostly at home and a different one everywhere else. In those cases, during the early years, primary caregivers are the main source of language experience in one of the languages, but when children enter daycare, kindergarten, or school, peers and the academic environment increasingly impact children's language abilities in the prevalent community languages (Nicoladis & Genesee, 1997).

Caregivers often face choices with respect to which language(s) they will speak to their infant, as many bilingual infants have caregivers who are also bilingual. In many societies, monolingualism in the national language is normative (Hobsbawm, 1990), and bilingualism is portrayed as the exception. Consequently, many families who speak a heritage language feel pressure to assimilate, and thus may choose not to transmit their heritage language, in the hope that this will bring social and economic advantages for their children (Kutlu & Kircher, 2021). However, if children grow up without developing competence in their family's heritage language, this can have detrimental long-term effects: they may feel embarrassed about not being able to communicate with all family members; they may feel less connected to their cultural identity; and they may be upset with their parents for not supporting their heritage language development (De Houwer, 2021; Nakamura, 2020). By contrast, if children do develop competence in their heritage language, this positively impacts their healthy identity development as well as their well-being—and it has positive effects on family dynamics and interpersonal relationships among family members (Müller et al., 2020) while also increasing children's cultural knowledge.

In some cases (e.g., Quebec and Catalonia), multiple community languages are spoken, giving children more opportunities to learn both languages. However, in most societies, there is one language that is widely spoken in the community and has a higher sociolinguistic status. The higher-status community language is usually the one that is learned in school and is more prevalent in the media. Language status contributes to language attitudes (Giles & Watson, 2013), and historically, minority and heritage language speakers have tended to prioritize their children's acquisition of such higher-status community languages (e.g., Baker, 1992). Lower-status heritage languages are more difficult to maintain, as bilinguals learning them have fewer opportunities to hear and practice such languages, they tend to have fewer highly proficient speakers to interact with, and there is less societal support (Gathercole & Thomas, 2009). However, if parents consider a particular language to hold important social meaning because it represents their social identity and evokes ingroup loyalty, they are significantly more likely to transmit this language to their children—seemingly irrespective of its status (Kircher, 2019).

## Assessing Bilingual Development

When all languages are considered, bilinguals reach language milestones on a similar timetable as monolinguals, such as producing their first words, beginning to combine words, and learning various syntactic forms (e.g., Nicoladis and Genesee, 1997). However, there are some differences between infants learning one and multiple languages, which means that their language development path can look different when these two groups are compared directly.

One example is vocabulary development. When measuring bilingual infants’ total vocabulary (i.e., counting words known in both languages), they know the same number of words as their monolingual peers (and sometimes even more). However, when measured separately, the number of words bilingual infants understand and produce in each of their languages is tightly coupled with their experiences with those languages, especially in word production (Thordardottir, 2011). That is, an infant hearing a language 30% of the time would be expected to know fewer words in that language than an infant hearing that language 60% of the time, who in turn would know fewer words than an infant hearing it 100% of the time—keeping in mind that bilinguals will get complementary exposure and learn additional words in their other language(s) (Côté, et al., 2021; De Houwer et al., 2014; Hoff, et al., 2012).

In some cases, differences in the trajectory of indicators such as vocabulary size have been the source of misdiagnoses—adding to the myths around bilingualism in infancy. For example, if a clinician screens children for language delay based on the English words they know, a child who hears English 30% of the time and Spanish 70% of the time could be flagged because their English word knowledge might be low compared to monolinguals, even though it is typical for an infant with that pattern of bilingual exposure (the child will likely know many additional words in Spanish, which was not measured). Fortunately, researchers are increasingly developing measures and strategies for more appropriately assessing bilingual children (e.g., Gampe et al., 2018; Peña et al., 2018). On the other hand, while bilingualism itself does not cause language disorders or delays (Paradis et al., 2011), real language difficulties can sometimes be ignored when children are bilingual. For example, a clinician may notice that a bilingual child's development is behind that of monolingual peers, and could attribute that solely to bilingualism, thus missing a language disorder if one was present.

## Implications for Policy

Having provided an overview of key research results regarding the development of young bilinguals, we now turn to their implications for policy (for a Figure summarizing the key points see supplemental materials Fibla, et al., 2021).

### Caregivers and Communities Need Adequate Support to Raise Children Bilingually

Bilingual experience in the early years is critical for bilingual language development, and young children possess the capacity to acquire multiple languages simultaneously. Parents, caregivers, and communities should be empowered to raise children with multiple languages from infancy if they so desire. Awareness should be raised of the long-term, positive consequences of the intergenerational transmission especially of heritage languages—not only for the child but also for the family and culture as a whole. Public policy interventions have the power to help change language attitudes (e.g., Hawkey, 2018; Kircher, 2014). Moreover, since language and culture go hand in hand, positive attitudes toward heritage languages might also increase positive attitudes towards minoritized groups (e.g., Indigenous peoples, migrants). Public support for bilingualism will allow families to navigate child-rearing in dynamic ways that match their goals for their children, while integrating biculturalism in societies.

### High-quality Interactions Between Caregivers and Children Should Be Promoted

High-quality, playful interactions between caregivers and infants should be promoted. In many bilingual families, these interactions often include code-switching, which should be understood as a normal part of bilingual communication and neither be discouraged nor encouraged. One mechanism to support high-quality bilingual interactions is to promote shared book-reading practices at home in the family's multiple languages. To do so, bilingual families need access to books in each of their languages, particularly in heritage languages, and bilingual books. Storybooks Canada (Stranger-Johannessen et al., 2018) is an example of a free online resource for culturally diverse stories with text and/or audio in over 30 languages, including many Indigenous and immigrant heritage languages.

### Paid Parental Leave Can Help Primary Caregivers Provide High-quality Language Experiences to Their Bilingual Infants

Because frequent, high-quality language experience is essential for early language learning, young bilinguals’ development will benefit from policies that allow key caregivers to spend more time with infants during the first years of life, if they desire. Studies with monolingual children highlight that paid family leave, particularly maternal leave after birth, can lead to better language outcomes during toddlerhood (Kozak et al., 2021) and help decrease sociodemographic health disparities, thus resulting in healthier neurocognitive development in infancy (Brito et al., 2021). Countries such as Canada and Sweden offer policies that allow families with two parents to share the total amount of leave, which can help ensure that their infants get high-quality exposure from multiple caregivers in multiple languages.

### Some Bilingual Children Need Extra Support, Including Those Learning Heritage Languages

Children learning languages that are spoken less widely in the community, such as speakers of Indigenous and immigrant heritage languages, need extra support to foster high-quality experiences in their languages. Many infants and toddlers spend considerable time in nonfamilial childcare—for example with nannies or in daycare and kindergarten. The availability of childcare in different languages can help support families’ language choices and children's development. Tailored preschool programs can be developed that not only expose children to diverse languages, but promote positive attitudes and related cultural knowledge. For example, the “Sacred Little Ones” program (Aziz-Parsons, 2017) develops early childhood education projects tailored to Native American communities in the United States, promoting both languages and cultures. Institutions such as libraries and cultural centers can provide useful supports, such as children's books in different languages and in bilingual formats, and multilingual storytime, whether in-person, recorded, or live-streamed (for examples see Association of Americans & Canadians in Israel, 2012; National Library Board, 2021; New York Public Library, 2021). Such community programs can also create and support social networks among families that share the same language(s).

### Bilingualism Needs to Be Supported in Typically and Atypically Developing Children

Both typically and atypically developing children grow up bilingual, and all such children should receive support in relation to their needs. Therefore, early education policies should explicitly address bilingual language learning, including addressing bilingual children with special needs (Pesco et al., 2016). Bilingual children benefit when they have access to clinical and educational services and support in all of their languages. Consequently, training on bilingualism is needed for educational and healthcare professionals. Historically, professionals have often recommended to bilingual families that they should only use one language when raising children with developmental disorders. However, such recommendations are not evidence-based and can decrease both the amount of high-quality speech infants hear and opportunities to practice conversational skills (Davis et al., 2021; Kaiser et al., 2001). Further, the inability to communicate in a heritage language may exclude the child from family culture and values (Kay-Raining Bird et al., 2012).

In evaluating whether bilingual children have language disorders or delays, they should not be assessed in the same way as monolinguals because most tools do not take into account the bilingual child's full, diverse language achievements. Whenever possible, evaluations should be conducted by trained professionals who are knowledgeable about bilingual assessment, and if possible fluent in each of the child's languages (Nayeb et al., 2021). Moreover, services should be provided in each of the child's languages. This goal can be supported by encouraging communication between families, school districts, clinicians, and universities that train speech-language therapists.

### Each Bilingual Family and Community Is Unique and Has Different Needs

Policies need to take into account variability across families and communities so that they can accommodate each bilingual child's profile. Policymakers should take time to understand the community of interest, and take into account the unique linguistic, cultural, and societal circumstances of the specific community. In this way, policymakers can develop tailored ways of promoting bilingual proficiency *in context*.

## Conclusion

Many infants around the world grow up learning more than one language, but raising bilingual children is not always easy. Families need both individual and societal support in promoting positive attitudes towards bilingualism and biculturalism, and providing children with sufficient opportunities to hear and use their different languages. Effective policies have the potential to support families, clinicians, and educational professionals to base decisions on the ever-growing science of bilingualism. Because every bilingual experience is different, policies that aim to support bilingual language learning should take into account each family context as well as their sociocultural environment.

Integrating bilingualism into our social fabric will ensure that children have the opportunities and advantages that come with learning more than one language. This will only be possible if we develop policies that support bilingual learning beginning in infancy. To inspire new ideas, an important best practice is to consult directly with members of the community in order to understand their goals, needs, and values—from their perspective and using their own words.
